# COVID-19 Vaccination Acceptance and Its Associated Factors among Cancer Patients in Tunisia

**DOI:** 10.31557/APJCP.2021.22.11.3499

**Published:** 2021-11

**Authors:** Houyem Khiari, Ines Cherif, Fehmi M’ghirbi, Amel Mezlini, Mohamed Hsairi

**Affiliations:** 1 *Department of Epidemiology and Biostatistics, Salah Azaiz Institute, Tunis, Tunisia. *; 2 *Department of Medical Oncology, Salah Azaiz Institute, Tunis, Tunisia. *

**Keywords:** COVID-19 vaccines, vaccine acceptance, attitudes, neoplasms, Tunisia

## Abstract

**Background:**

Vaccination is the most effective way to fight the COVID-19 pandemic and to protect people who have a higher risk of developing severe illness and death from COVID-19 such as cancer patients. We aimed in this study to determine the acceptance rate of COVID-19 vaccination of the Salah Azaiez Institute (SAI) of cancer of Tunisia patients and to identify its associated factors.

**Methods:**

It was a cross sectional study about patients admitted to the SAI for treatment during the month of February 2021. Univariate and multivariate analyses were performed to identify factors associated with the COVID-19 vaccine acceptance among Tunisian cancer patients.

**Results:**

A total of 200 patients were included in this study with a mean age of 54.4±12.7 years and a gender ratio of 0.5. Only 35.0% of surveyed patients reported their acceptance to receive the COVID-19 vaccine. Multivariate analysis showed that believing in COVID-19 vaccine safety and efficacy (OR=3.1 [1.3-7.4]), enrollment in the COVID-19 vaccine platform (OR=8.3 [1.8-38.1]) and the willingness to receive influenza vaccine (OR=3.9 [1.6-9.3]) were independently associated with the COVID-19 vaccine acceptance among SAI cancer patients.

**Conclusions:**

The COVID-19 vaccine acceptance rate found in this study was low. Communication strategies of the vaccination campaigns should provide clear, simple and detailed messages about the efficacy and the benefits of the COVID-19 vaccines. More engagement of health authorities to promote COVID-19 vaccination is necessary.

## Introduction

The coronavirus disease 2019 (COVID-19) is the worst pandemic of the twenty-first century in terms of morbidity and mortality (Feehan and Apostolopoulos, 2021). The World Health Organization (WHO) declared on 30 January 2020 the novel coronavirus as ‘Public Health Emergency of International Concern’ (Nueangnong et al., 2020).

Globally, the severe acute respiratory syndrome coronavirus 2 (SARS-CoV-2) has caused more than 2.6 million deaths in February 2021 against 1.7 million deaths in December 2020 (Roser et al., 2020).Thus, SARS-CoV-2 infectivity still continue to spread across the globe and the epidemiological situation is changing rapidly (Madahar et al., 2021). As viruses mutes rapidly, several new variants of SARS-CoV-2 have already been identified in South Africa (Tegally et al., 2020), in the United Kingdom (Kirby, 2021; Volz et al., 2021b) and in Brazil (Nonaka et al., 2021; Toovey et al., 2021) and have emerged across the globe (WHO,2020a). Moreover, these variants spread more easily and infect more people than other variants (Tegally et al., 2020; Du et al., 2021; Volz et al., 2021a).

According to the WHO and Centers for Disease Control and Prevention (CDC), people who are older than 60 years or who have certain health conditions such as hypertension, diabetes, cardiovascular diseases, chronic respiratory diseases, weakened immune systems and cancer have higher risk of severe illness and mortality of COVID-19 (WHO, 2020b; CDC, 2021). Data from several sources, including several meta-analyses, suggest that developing severe illness and death from COVID-19 is higher among patients with cancer (Afshar et al., 2020; Curigliano, 2020; Dewi et al, 2020; Lee et al., 2020; Saini et al., 2020; Ferrari et al., 2021; Yang et al., 2021; Zarifkar et al., 2021). Recommendations of the National Comprehensive Cancer Network (NCCN) COVID-19 Vaccination Advisory Committee mentioned that patients with active cancer and those on treatment should be prioritized for vaccination (Hwang et al., 2021; Ribas et al., 2021). Hence, adherence to preventive measures and vaccination are essential to mitigate the rapid spread of the virus. Adherence to these measures is directly affected by knowledge, attitude, and practice of patients towards COVID-19. 

Previous pandemics showed relatively high vaccine hesitancy and relatively low vaccination coverage (Seale et al., 2010; Chor et al., 2011; MacDonald, 2015). Indeed, WHO listed vaccine hesitancy as one of the top 10 threats to global health in 2019 (Thangaraju and Venkatesan, 2019). Vaccine hesitancy about COVID-19 has already caused concern, especially as herd immunity for COVID-19 requires an estimated 55% to 82% vaccine uptake (DeRoo et al., 2020) and despite the proved safety and efficacy of most Covid-19 vaccines (Xing et al., 2021), scepticism about the vaccines persists (Lazarus et al., 2021; Sallam, 2021; Schwarzinger et al., 2021).

In Tunisia, in less than a year since the first confirmed case of COVID-19 reported on March 2, 2020 more than 242,000 cases and 8404 deaths by COVID-19 have been recorded. Assuming that generalized vaccination of the population is not possible for now, the national vaccination strategy, will first target high risk population groups, starting with persons aged 60 years or above, health care workers and patients with chronic diseases such as cancer. But, few people enrolled in the COVID-19 vaccination platform to receive the vaccine so far. 

To our knowledge, no previous studies have assessed COVID-19 vaccine acceptance in Tunisia and in the North African region. Thus, the present study aimed to determine the acceptance rate of COVID-19 vaccination of the Salah Azaiez Institute (SAI) patients and to identify its associated factors. 

## Materials and Methods

*Study Design and population*

This was a cross sectional study conducted in the SAI of cancer which constitute the reference center for diagnostic and treatment in Tunisia. All patients above 18 years of age, who were admitted to the hospital for treatment during the month of February 2021 who were able and accepted to respond the questionnaire were included in the study.

*Study Instrument*

We used a well-structured questionnaire composed of two sections: the first one included questions on socio-demographic characteristics of the study population (age, gender, educational level, profession). The second part was related to Vaccine literacy (VL), perceptions and behavior towards COVID-19 vaccination (Biasio et al., 2020a). VL level was assessed using questions related to functional skills (semantic system) and interactive-critical skills (cognitive efforts). Responses were rated according to a 4-point Likert scale (4 – never, 3 – rarely, 2 – sometimes, 1 – often). This test was validated and used by previous studies (Ishikawa et al., 2008; Aharon et al., 2017; Del Giudice et al., 2018; Biasio et al., 2020a).

Attitudes and behaviors were assessed via closed questions (yes/no). To evaluate patients’ beliefs about vaccinations, two more questions were asked (‘’I am not favorable to vaccines because they are unsafe’’ and ‘‘There is no need to vaccinate, because natural immunity exists’’, considered as ordinal variables). Answers were rated according to a four-point Likert scale (Strongly agree, Agree, Disagree, Strongly disagree).The original questionnaire (Biasio et al., 2020a; Biasio et al., 2020b) was translated from English into Arabic language by a bilingual translator, and then back-translated into English by another bilingual translator. Translation and back-translation were harmonized by the research team.

*Data Collection*

Data were collected by one medical investigator, who conducted a face to face interview with inpatients of the SAI during the month of February 2021. All interviewed patients were clearly informed about the purpose of the study.

*Data Analysis*

Data were analyzed using the software SPSS version 26. Categorical variables were expressed as percentages and quantitative variables as means and standard deviations.

Functional and interactive-critical VL scores were obtained from the mean value of the responses to each scale (range from 1 to 4), a higher value correspond to a higher VL level.

Cronbach’s alpha coefficient was used to measure the internal consistency of the VL scales. Chi square and Student’s T test were used respectively for the comparison of percentages and means. The significance level was set at p <0.05. 

A multivariate logistic regression analysis was performed to determine factors associated to the acceptance to get COVID-19 vaccination. To facilitate the interpretation of results of multivariate analysis, answers to questions on patients’ beliefs about vaccinations were dichotomized by grouping “Strongly agree” with “Agree” into “Agree” and “Strongly disagree” and “Disagree” into “Disagree”. Functional and interactive-critical VL scores were divided into two classes according to the median to obtain qualitative variables. “I don’t know” responses were excluded from univariate and mutivariate analysis as their frequency didn’t’ exceed 10%.

*Ethical considerations*

Ethical approval was obtained from the Ethical Committee of the SAI. An informed verbal consent was taken from the participants before the interview. We also respected anonymity while collecting data. 

## Results

Two hundred patients were included in this survey with a mean age of 54.4±12.7 years ranging from 19 to 81 years and a gender ratio of 0.5 (34.5% of males). The majority of participants (71.0%) had a primary or secondary level of education and one fifth were illiterate. Around half of the asked patients (50.5%) were unemployed and 5.0% were healthcare workers. Nearly the half of participants (43.5%) had a history of chronic diseases and only 2.0% had a history of confirmed COVID-19 infection (Table 1). 

**Figure 1:**
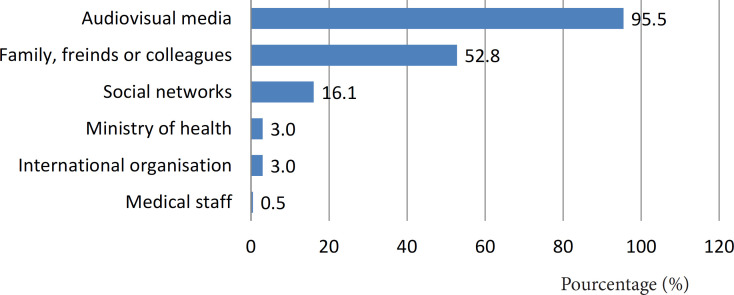
Most Consulted Information Sources on COVID-19 Vaccination

One quarter of the asked patients didn’t know how to register to get the COVID-19 vaccine and only 9.0% enrolled in the registration platform. Information sources most frequently used by the respondents were TV and radio (95.5%) followed by the entourage (52.8%) (Figure 1).

*Vaccine literacy*

Cronbach’s alpha coefficient of the VL scales were respectively of 0.89 for functional skills and 0.78 for interactive skills showing a good internal consistency. Mean score of VL was significantly associated with the acceptance to get the COVID-19 vaccine with respectively 2.6±0.8 for yes answers and 2.3±0.7 for no (p=0.02). This association was essentially in relation with Interactive VL score (p=0.001). The average functional VL score was high of 3.2±1.0, while the interactive-critical score was low of 1.7±0.9 out a maximum of 4. Higher functional and interactive VL scores were significantly associated with higher educational level (p<10-3). Unlike functional VL, interactive score was significantly associated to the occupation (p<10-3) (highest interactive VL score was among healthcare workers and lower one was among unemployed patients).There was no significant association between age and gender with both interactive and functional scores (Table 2).

*Attitudes towards COVID-19 vaccines*

Only 35.0% of surveyed patients reported their acceptance to get the COVID-19 vaccine and 21.0% were ready to pay a fee to get the vaccine. Majority of patients (74.0%) believed that COVID-19 vaccines are unsafe and ineffective and that health authorities will not be able to vaccinate the majority of the Tunisian population.

*Behavior regarding current vaccinations*

The proportion of willingness to get Influenza vaccine was close to the COVID-19 one with a percentage of 39.0%. However, willingness to be vaccinated against other infectious diseases was much higher (69.0%) (Table 3).

*Beliefs towards vaccinations*

Regarding vaccination in general, nearly half of patients (42.0%) were not favorable to vaccines because they are unsafe and three quarter (74.5%) thought that there is no need to vaccinate because natural immunity exists (Table 3). 

*Factors associated to the willingness to receive COVID-19 vaccine*

According to this study, socio-demographic characteristics were associated to the willingness to get COVID-19 vaccine except for the educational level. The association was more significant among males (OR=2.2), patients aged 60 years or over (OR=7.5) and retired patients (OR= 3.7). Concerning VL, the interactive skills score was very associated with the acceptance of the COVID-19 vaccination (interactive score= 3.1[1.5-6.2]; OR=3.1). However, no significant association was found between the acceptance to get the vaccine and the functional skills score. Patients who believed that COVID-19 vaccine is safe and effective and those who accepted to get influenza vaccination were significantly more ready to receive COVID-19 vaccine with respectively an OR of 4.5 and 4.9.

In multivariate analysis, believing in COVID-19 vaccine safety and efficacy (p=0.011; OR=3.1 [1.3-7.4]), enrollment in the COVID-19 vaccine platform (p=0.007; OR=8.3 [1.8-38.1]) and the willingness to receive influenza vaccine (p=0.002; OR=3.9 [1.6-9.3]) were independently associated with COVID-19 vaccine acceptance among SAI cancer patients.

**Table 1:** General Characteristics and Medical History of Participants

Variables	Number (%)
Gender	
Male	69 (34.5)
Female	131 (65.5)
Age (years)	
19-40	30 (15.0)
40-60	101 (50.5)
>60	69 (34.5)
Educational level	
Illiterate	41 (20.5)
Primary school	68 (34.0)
High school	74 (37.0)
University degree	17 (8.5)
Occupation	
Healthcare workers	10 (5.0)
Worker	51 (25.5)
Retired	36 (18.0)
Unemployed	103 (51.5)
Chronic diseases	
Hypertension	45 (22,5)
Diabetes	33 (16,5)
Obesity	9 (4,5)
Heartdiseases	6 (3,0)
Asthma	4 (2,0)
No	114 (57.0)
History of confirmed COVID-19 infection	
Yes	4 (2.0)
No	196 (98.0)

**Table 2:** Association between Vaccine Literacy Scores and Socio-Demographic Characteristic and Acceptance to be Vaccinated by COVID-19 Vaccine of the Studied Population

VL	Characteristics		Mean ± SD	P
Functional skills	Age groups	[19 - 40]	3,3±0,9	0.64
		[40 - 60]	3,2±0,9	
		>60	3,1±1,0	
	Gender	Male	3.2±1.0	0.84
		Female	3.2±1.0	
	Educational level	Illiterate	2.5±1.2	<10-3
		Primary school	3.3±1.0	
		High school	3.4±0.9	
		University degree	3.7±0.5	
	Occupation	Healthcare workers	3.8±0.5	0.07
		Other occupation	3.3±1.1	
		Retired	3.4±0.9	
		Unemployed	3.1±1.1	
	Willingness to get the COVID-19 vaccine	Yes	3,2±1,0	0.70
		No	3,1±1,0	
Interactive skills	Age groups	19 -40	1,6±0,7	0.46
		40 - 60	1,8±0,9	
		>60	1,8±1,0	
	Gender	Male	1.9±0.9	0.12
		Female	1.7±0.8	
	Educational level	Illiterate	1.3±0.5	<10-3
		Primary school	1.6±0.8	
		Highschool	1.8±0.9	
		University degree	2.8±1.0	
	Occupation	Healthcare workers	2.5±1.3	<10-3
		Other occupation	1.9±1.1	
		Retired	1.9±0.9	
		Unemployed	1.5±0.7	
	Willingness to get the COVID-19 vaccine	Yes	2,0±0,9	0.001
		No	1,5±0,8	

**Table 3:** Attitudes, Behavior and Beliefs toward COVID-19 Vaccine

Attitudes towards COVID-19 vaccine	Number (%)
Acceptance to get the COVID-19 vaccine	
Yes	70 (35.0)
No	125 (62.5)
Don't know	5 (2.5)
COVID-19 vaccine is safe and effective	
Yes	52(26.0)
No	148 (74.0)
Paying a fee to get the COVID-19 vaccine	
Yes	42 (21.0)
No	158 (79.0)
Health authorities will be able to vaccinate the majority of Tunisian population	
Yes	71(35.5)
No	112(56.5)
I don’t know	16(8.0)
Behavior regarding current vaccinations	Number (%)
Influenza vaccine uptake in the last influenza season	
Yes	13 (6.5)
No	187 (93.5)
Willingness to get the influenza vaccine	
Yes	78 (39.0)
No	121 (60.5)
I don’t know	1 (0.5)
Willingness to be vaccinated against other infectious diseases	
Yes	138 (69.0)
No	62 (31.0)
Beliefs regarding vaccination in general	Number (%)
I am not favorable to vaccines because they are unsafe	
Totally agree	22 (11.0)
Agree	62 (31.0)
Disagree	60 (30.0)
Totally disagree	54 (27.0)
I don’t know	2 (1.0)
There is no need to vaccinate because natural immunity exists	
Totally agree	83 (41.5)
Agree	66 (33.0)
Disagree	27 (13.5)
Totally disagree	20 (10.0)
I don’t know	4 (2.0)

**Table 4:** Univariate and Multivariate Analysis: Acceptance to get the COVID-19 vaccine by participants’ socio-demographic characteristics, Knowledge, attitudes and behaviors regarding vaccines

Univariate analysis Factors		Willingness to receive COVID-19 vaccineN(%)		Crude OR [95% CI]		P value	
socio-demographic characteristics							
Gender							
Female		37 (28.9)		1		0.008	
Male		33(47.8)		2.2 [1.2-4.1]			
Age category based on quartiles							
19-40		3(10.3%)		1		0.003	
40-60		35 (35.4)		4.7[1.3-16.8]			
>60		32 (46.4)		7.5 [2.1-27.1]			
Educational level							
Illiterate		13 (33.3)		1		0.490	
Primary school		28(41.2)		1.4 [0.6-3.3]			
Secondary school		22(30.1)		0.9[0.4-2.0]			
University degree		7(43.8)		1.6 [0.5-5.3]			
Occupation							
Unemployed		30 (29.4)		1		0.004	
Healthcare workers		4 (44.4)		1.9 [0.5-7.5]			
Retired		22 (61.1)		3.7 [1.7-8.3]			
Other occupations		14 (28.0)		0.9 [0.4-1.9]			
Chronic diseases							
No		32 (28.8)		1		0.019	
Yes		38 (44.7)		2.0[1.1-3.6]			
Knowing about how to register to get the COVID-19 vaccine							
No		14 (28.0)		1		0.198	
Yes		56(38.1)		1.6[0.8-3.2]			
Registration in the COVID-19 vaccine platform							
No		57(31.7)		1		<10-3	
Yes		13 (76.5)		7.0[2.2-22.5]			
Knowledge about COVID vaccine							
VL: Functional skills score							
1.0-2.5		18 (32.7)		1		0.610	
2.5-4.0		52 (36.6)		1.2 [0.6-2.2]			
VL: Interactive skills score							
1.0-1.4		27(27.3)		1		0.015	
1.4-4.0		43 (43.9)		2.1[1.1-3.8]			
Attitudes towards COVID-19 vaccine							
COVID-19 vaccine is safe and efficacious							
Other responses		38(26.2)		1		<10-3	
Yes		32 (61.5)		4.5[2.3-8.8]			
Health authorities will be able to vaccinate the entire population							
No		32 (29.1)		1		0.037	
Yes		31 (44.3)		1.9 [1.0-3.6]			
Behavior regarding current vaccinations							
Influenza vaccine uptake in the last influenza season							
No		62 (33.7)		1		0.043	
Yes		8 (61.5)		3.1[0.9-10.0]			
Willingness to receive the influenza vaccine							
No		26 (21.7)		1		<10-3	
Yes		43 (57.3)		4.9 [2.6-9.1]			
Factors		Willingness to receive COVID-19 vaccineN(%)		Crude OR [95% CI]		P value	
Beliefs regarding vaccination						
I am not favorable to vaccines because they are unsafe						
Agree	12 (14.6)		1		<10-3	
Disagree	57 (50.4)		5.9[2.9-12.1]			
There is no need to vaccinate because natural immunity exists						
Agree	40 (27.2)		1		<10-3	
Disagree	28 (60.9)		4.2[2.1-8.3]			
Factors	Adjusted odds ratio					
Multivariate analysis	Adjusted odds ratio				P value	
COVID-19 vaccine is safe and efficacious						
Other responses	1				0.011	
Yes	3.1 [1.3-7.4]					
Enrollment in the COVID-19 vaccine platform						
No	1				0.007	
Yes	8.3 [1.8-38.1]					
Willingness to receive the influenza vaccine						
No	1				0.002	
Yes	3.9 [1.6-9.3]					

## Discussion

The present study aimed to determine the acceptance rate of COVID-19 vaccine among cancer patients of the SAI and to identify its associated factors. Results of this study revealed a low acceptance rate to get the COVID-19 vaccine (35.0%) among cancer patients of the SAI of Tunisia. Multivariate analysis showed that believing in COVID-19 vaccine safety and efficacy (p=0.011; OR=3.1 [1.3-7.4]), the willingness to receive influenza vaccine (p=0.002; OR=3.9 [1.6-9.3]) and the enrollment in the COVID-19 vaccination platform (p=0.007; OR=8.3 [1.8-38.1]) were independently associated with the COVID-19 vaccine acceptance among SAI cancer patients.

This study is, to our knowledge, the first of its kind in Tunisia as well as in the North African region. It provides benchmark data to guide health authorities for improving the COVID-19 vaccination strategy in order to increase vaccine acceptance and uptake. However, the cross sectional study design does not allow to establish a causal relationship between the COVID-19 vaccine acceptance and the identified associated factors.

In comparison with other studies among cancer patients, the acceptance rate to get the COVID-19 vaccine among the studied patients (35.0%) was very low. In Romania, a study about knowledge attitude and practice towards COVID-19 reported that 72.2% of the studied oncological patients declared that they would get the coronavirus vaccine when available (Gheorghe et al., 2021). Another study among American cancer patients reported that 71% of participants had the intention to receive a COVID-19 vaccine (Kelkar et al., 2021). A lower percentage was reported in a French survey among a population with cancer, where 53.7% reported their intent to be vaccinated (Barrière et al., 2021).

In comparison to the general population, acceptance rate of COVID-19 vaccination of the present study is much lower than the rate of the majority of studies in the world with globally a rate of over 70 ; however, the reported rate in our study is quite similar to those detailed in some middle east countries such as in Kuwait (23.6%) and Jordan (28.4%) (Sallam, 2021). According to the literature, the highest COVID-19 vaccine acceptance rate was found in East and South East Asia with rates higher than 90% (Harapan et al., 2020; Wang et al., 2020) and lowest ones were described in the Middle East, Russia, Africa and several European countries (Italy:53.7;Poland:56.3% and France :58.9%) (Sallam, 2021).The proportion of willingness to get Influenza vaccine was close to the COVID-19 one with respectively 39.0% and 35.0%. These rates were similar to those reported in some Middle East countries among the general public in Jordan, Kuwait and other Arab countries where the average acceptance rates for COVID-19 and influenza vaccines were respectively of 29.4% and 30.9% (Sallam, 2021). In fact, the willingness to receive influenza vaccine and believing in COVID-19 vaccine safety and efficacy were independently associated with the COVID-19 vaccine acceptance in our study. These findings are consistent with several other studies where the main reasons for vaccination refusal or hesitancy were concerns about the efficacy of the COVID-19 vaccines and a lack of trust in them (Malik et al., 2020; Kaplan and Milstein, 2021). Mistrust in vaccines and their safety is usual when a new vaccine is developed but it can be induced, or amplified by many other factors such as the exposure to negative messages (misinformation, disinformation, conspiracy theories and fake news) and also negative opinions about vaccines (especially through the media or the family) (Kochhar and Salmon, 2020). Research have proved that negative messages are associated with decreases in vaccine confidence and uptake (Wu et al., 2015; Dunn et al., 2017; Larson et al., 2019; Gørtz et al., 2020; Hansen et al., 2020; Paul et al., 2021). Hence, several approaches were developed to overcome vaccine refusal such as opposing the spread of false information, providing a framework that could be used by healthcare providers to increase patients confidence in COVID-19 vaccines. The most associated independent factor to COVID-19 vaccine acceptance was the registration to get the vaccine. In fact, one quarter of the surveyed patients didn’t know about the possibility of enrollment to receive the COVID-19 vaccine and only 9.0% have registered to the platform. These results reflect the weakness of the Tunisian communication campaign to inform and sensitize the population about COVID-19 vaccination. Clear, simple and detailed messages about the efficacy and the benefits of the COVID-19 vaccines, ways of registration to get the vaccine and detailed instructions about the online platform of registration are recommended. Technical support should also be offered to disadvantaged categories (elderly, illiterate, low socio-economic level...).

High rates of negative attitudes were reported in this study, as three quarter of patients believed that COVID-19 vaccines are unsafe and ineffective and that health authorities will not be able to vaccinate the majority of the Tunisian population. This reflect a serious lack of confidence in the vaccine itself but also in policy-makers and the health system (Larson et al., 2015; Larson et al., 2018; Basu P, 2020). According to the WHO, key determinants of trust on health system are the way that people perceive the competence of health authorities, the objectivity of the provided information and actions, the consistency between their messages and actions and their sincerity by showing transparency and empathy through actions (WHO, 2017). Policy makers should be engaged to combat mistrust and build public confidence, which is one of the key solutions for vaccine hesitancy. In addition, the present study assessed the level of VL of the surveyed population and showed that the mean functional VL score was high, however the interactive-critical one was very low in comparison with the literature (Biasio et al., 2020a). Thus, the studied population seems to have sufficient skills in reading and comprehending COVID-19 vaccine information ; however their ability to be actively involved to make their own decisions about COVID-19 vaccination was weak (Lorini et al., 2018). Moreover, univariate analysis showed that VL average score was associated to the acceptance to get the COVID-19 vaccine. Some studies reported similar results regarding influenza vaccination and found a significant positive relationship between HL and vaccination uptake (White et al., 2008; Bennett et al., 2009; Lorini et al., 2018). A recent systematic review which aimed to comprehend the role of HL as a determinant of vaccine hesitancy mentioned that few studies aimed to assess this scope especially in low and middle income countries (LMIC) and that the role of HL in predicting vaccine hesitancy or acceptance could be influenced by various factors (country, age, type of vaccine) (Lorini et al., 2018).Thus, communication strategies about COVID-19 vaccination should build VL to redress vaccine hesitancy (Lazarus et al., 2021). 

In conclusion, the low rate of acceptance of COVID-19 vaccines, and related negative attitudes reported in the current study underscores the need to strengthen communication strategies of the COVID-19 vaccination campaigns. Involving the civil society organizations in the promotion of the vaccines against SARS-CoV-2 may also be helpful. Healthcare workers play key role in sensitizing high risk patients to receive the COVID-19 vaccine. A strong health authority’s engagement is recommended to provide people who are hesitant, distrusting or unmotivated to the COVID-19 vaccines with needed resources, information and support to help them make the right decision about the vaccine acceptance and uptake.

## Author Contribution Statement

Conceptualization, methodology : KH ; Data collection, Statistic analysis: IC and FM ; Writing: IC, KH and FM ; Review, Editing: HM, AM and KH. All authors read and agreed with the published version of the manuscript. 

## Ethical approval

This research was approved by the Ethics Committee of the SAI.

## Conflict of interest

All authors declare no conflict of interest.
